# Preparation of High Mechanical Performance Nano-Fe_3_O_4_/Wood Fiber Binderless Composite Boards for Electromagnetic Absorption via a Facile and Green Method

**DOI:** 10.3390/nano8010052

**Published:** 2018-01-21

**Authors:** Baokang Dang, Yipeng Chen, Hanwei Wang, Bo Chen, Chunde Jin, Qingfeng Sun

**Affiliations:** 1 School of Engineering, Zhejiang A&F University, Hangzhou 311300, China; dang972028790@163.com (B.D.); 18868195633@sina.cn (Y.C.); 18868196590@163.com (H.W.); jincd@zafu.edu.cn (C.J.); 2 Zhejiang New Wood Material Technology Co., Ltd., Ningbo 315300, China; cb19871001@126.com

**Keywords:** wood fiber composites, Fe_3_O_4_ nanoparticles, electromagnetic absorption properties, mechanical properties

## Abstract

Fe_3_O_4_/wood fiber composites are prepared with a green mechanical method using only distilled water as a solvent without any chemical agents, and then a binderless composite board with high mechanical properties is obtained via a hot-press for electromagnetic (EM) absorption. The fibers are connected by hydrogen bonds after being mechanically pretreated, and Fe_3_O_4_ nanoparticles (NPs) are attached to the fiber surface through physical adsorption. The composite board is bonded by an adhesive, which is provided by the reaction of fiber composition under high temperature and pressure. The Nano-Fe_3_O_4_/Fiber (NFF) binderless composite board shows remarkable microwave absorption properties and high mechanical strength. The optional reflection loss (RL) of the as-prepared binderless composite board is −31.90 dB. The bending strength of the NFF binderless composite board is 36.36 MPa with the addition of 6% nano-Fe_3_O_4_, the modulus of elasticity (MOE) is 6842.16 MPa, and the internal bond (IB) strength is 0.81 MPa. These results demonstrate that magnetic nanoparticles are deposited in binderless composite board by hot pressing, which is the easiest way to produce high mechanical strength and EM absorbers.

## 1. Introduction

Many kinds of electronic products are used in daily life, such as mobile phones, televisions, computers, the internet and radar systems [1,2]. These products bring great convenience, but they inevitably increase the impact of electromagnetic (EM) interference pollution. Indoor EM interference damages human health. To reduce the problems with EM interference, EM absorbers were investigated to absorb unwanted EM signals and obstruct barriers made of conductive or magnetic materials [3,4]. The absorber attenuates electromagnetic energy by means of dielectric loss or magnetic loss, reflection or absorption of the radiation, and multiple reflection [5,6]. To solve EM interference, microwave absorbing materials were investigated for EM shielding. Fe_3_O_4_ nanoparticles were studied as the magnetic EM absorbers due to their superior mechanical, magnetic and dielectric properties, high compatibility and low toxicity, and strong spin polarization at room temperature [2,7]. Kong et al. investigated thermoplastic rubber filled with Fe_3_O_4_ nanocomposites, and the reflection loss was −25.51 dB when the filler content was 12 wt % [8]. Yang et al. prepared a graphene and Fe_3_O_4_ hybrid by chemical deposition, and the materials had a saturation magnetization of 4.62 emu/g [9]. The magnetic particles that fell off led to a decreased shielding effect, such that the magnetic nanoparticles (NPs) could be loaded into the composite. Considering market applications and the cost of EM shielding materials, biomass materials could be considered a matrix of EM-absorbing materials.

As the most abundant biomass materials, wood fibers exhibit many superior characteristics: low-cost, renewability and sustainability, biodegradability, low density, high strength and modulus [10,11]. Wood fiber is composed of cellulose, hemicellulose, lignin, pectin and some extractives [12]. Cellulose is present in the cell wall in the form of microfibers and nanofibers, which consist of crystalline and amorphous regions [13]. Under acid hydrolysis, cellulose shows a high modulus of 150 GPa and strength of 10 GPa due to the lack of chain folding, and they contain only a small number of defects [14,15]. The strength of cellulose is derived from hydrogen bonds and covalent bonds. The hydrogen bond is created via hydroxyl bonding of cellulose; the hydrolyzate of hemicellulose and the phenol hydroxyl of lignin produce a condensation reaction to form covalent bonds [16,17].

In this work, we report a Fe_3_O_4_/Fiber binderlelss composite board prepared by hot-pressing to have high mechanical strength and EM absorption in the presence of water. To combine Fe_3_O_4_ and wood fiber, a mechanical grinding pretreatment was carried out. Benefitting from the grinding pretreatment, Fe_3_O_4_ with narrow sizes uniformly distributed on the rough surface of the treated fiber. Compared to the modulus of rupture of pure binderless board, the binderless composite board was significantly improved by 43.4%. EM shielding was studied using complex permittivity and permeability [18]. The results indicated that the as-prepared materials displayed high mechanical strength and microwave absorption properties. The binderless composite board could be applied to indoor decoration materials to reduce indoor EM interference.

## 2. Materials and Methods

### 2.1. Materials

Wood fibers with average diameters of 40 μm were purchased from Zhejiang New Wood Material Technology Co., LTD. (Ningbo, China) Fe_3_O_4_ nanoparticles were provided by Shanghai Aladdin Biochemical Technology Co., Ltd. (Shanghai, China). Distilled water was used as the aqueous medium.

### 2.2. Synthesis of Nano-Fe_3_O_4_/Fiber Composite

First, 30 g wood fiber and 1500 mL distilled water were mixed, and then Fe_3_O_4_ NPs were added to the fiber suspension. The obtained mixed suspensions were subjected to mechanical grinding for 6 h (Model: JM-L80, Shanghai Shen’ou Valve Industry Co. Ltd. (Shanghai, China)) at 2880 rpm and the disc distance (0.15 mm) was kept constant. The fibers were delivered into the disc gap continuously through a loop consisting of a peristaltic pump and plastic tubing. The mass ratio of Fe_3_O_4_ NPs to fibers (m (Fe_3_O_4_)/m (fiber)) was set at 1%, 3%, 6% and 9%, and a series of nano-Fe_3_O_4_/Fiber (NFF) composites were coded as NFF1, NFF2, NFF3, and NFF4, respectively.

**Scheme 1:**
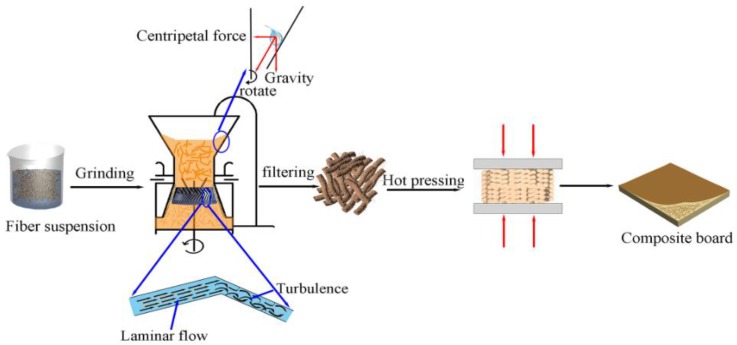
The preparation of the Nano-Fe_3_O_4_/Fiber (NFF) binderless composite board.

The mixed suspension was subjected to filtering and then assembled into a square model. The model was hot-pressed using a laboratory hot-press for 25 min, for which the pressure was 2.5 MPa at a temperature of 200 °C. After hot-pressing, the final products were named fiberboard, NFF1 board, NFF2 board, NFF3 board, NFF4 board. The process of NFF binderless composite board preparation is shown in Scheme 1.

### 2.3. Characterization

#### 2.3.1. Composite Structure

The micro-morphologies of fiber and Fe_3_O_4_/fiber composite were observed by scanning electron microscopy (SEM, Quanta FEG 250, Hillsborough, OR, USA) and transmission electron microscopy (TEM, Tecnai G2 F20 S-TWIN, Hillsborough, OR, USA). The element compositions and distributions were detected by energy dispersive X-ray spectroscopy (EDX, equipped in SEM). The crystal structures of samples were measured by X-ray diffraction (XRD, Bruker D8 Advance, Karlsruhe, Germany) with Cu Kα radiation (*λ* = 1.5418 Å). Fourier transform infrared spectroscopy (FTIR, Nicolet iN10 MX, Waltham, MA, USA) was measured the changes in chemical groups. The thermal stabilities were characterized by thermo-gravimetric analysis (TGA, STA449F3, Ahlden, Germany) with a heating rate of 20° min^−1^ under air atmosphere. X-ray photoelectron spectroscopy (XPS, Thermo ESCALAB 250XI, Waltham, MA, USA) detected the element compositions of Fe_3_O_4_/fiber composite.

#### 2.3.2. Electromagnetic Test

The electromagnetic properties of the magnetic composite boards were obtained by a vibrating sample magnetometer (VSM, Lake Shore 7307, Westerville, OH, USA). The electromagnetic permittivity values of the samples were measured in 2–18 GHz by a Keysight E5071C ENA vector network analyzer (Santa Clara, CA, USA).

#### 2.3.3. Mechanical Studies

The anti-bending mechanical properties and internal bonding (IB) strength of all binderless boards were measured by the three-point bending test, which used a universal testing machine with loading rate of 5 mm/min (MWD-100, Jinan, China). The samples had a dimension of 100 mm × 20 mm × 5 mm, and 15 test pieces were used for anti-bending mechanical properties. Fifteen specimens were cut into 50 mm × 50 mm × 5 mm, and used to measure the internal bonding (IB) strength. Fifteen samples with 50 mm × 50 mm × 5 mm were used to measure thickness swelling (TS), and immersed for 24 h in water at 20 °C. All experiments were measured in accordance with the National Standard GB/T 11718-2009 [19].

## 3. Results and Discussion

**Figure 1:**
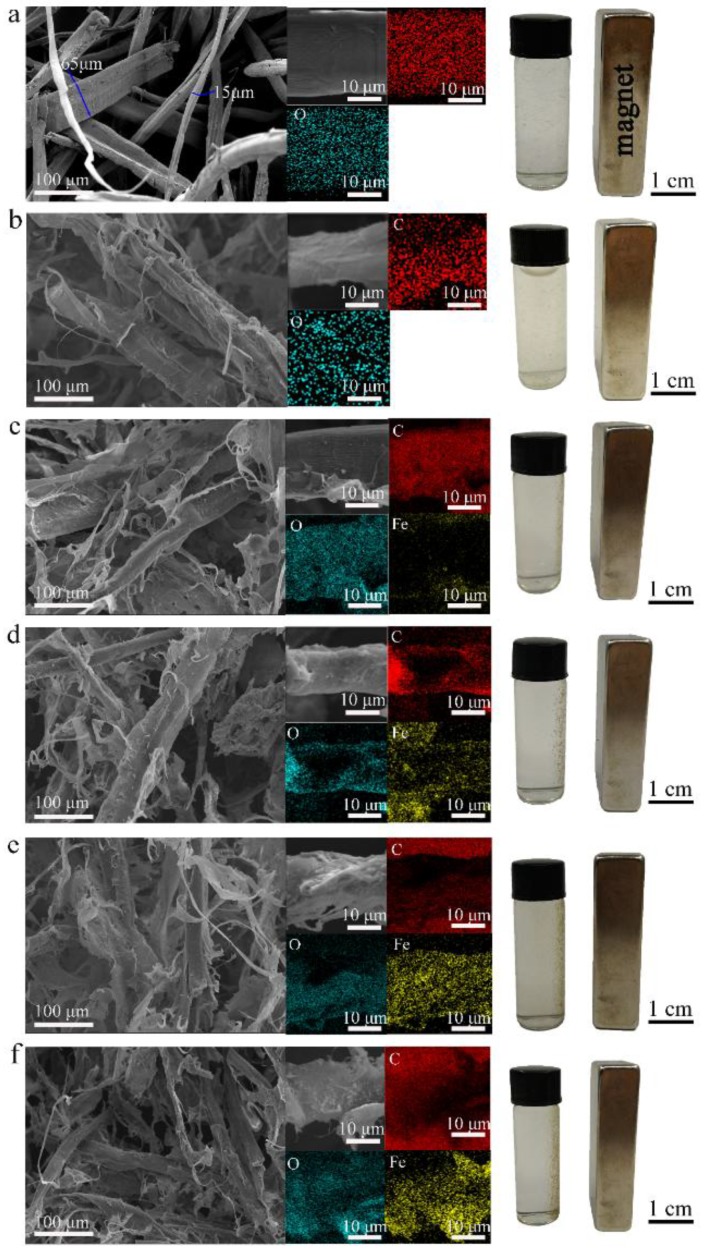
Digital images and scanning electron microscopy (SEM)/energy dispersive X-ray spectroscopy (EDS) mapping images of fiber (**a**); treated fiber (**b**) and nano-Fe_3_O_4_/Fiber (NFF) composite with different contents of Fe_3_O_4_ NPs. ((**c**) NFF1; (**d**) NFF2; (**e**) NFF3; (**f**) NFF4).

Figure 1 shows the micromorphology and EDX mapping images of treated fibers and NFF composites. The original fibers with an average diameter of 40 μm are displayed in Figure 1a, and the surfaces have a smooth structure. Figure 1b shows the morphology of the roughened surface of fibers after grinding. A small number of microfibers appeared on the fiber surface, and the diameter of the filament was approximately 600 nm. The macroscopic images of the original and treated fibers in Figure 1a,b demonstrated that the fibers did not have magnetic particles. The main element compositions of the original fiber and treated fiber were C and O, which were detected by EDX analysis. The micromorphology and macroscopic images of the NFF composite are shown in Figure 1c–f; the microfibers appeared on the fiber surface. This result was ascribed to the addition of NPs, which increased the friction between the fibers. The microfibers were cross-linked into a sheet structure, which was bonded via cellulose hydroxyl groups. Under high magnification, the fiber surface exhibited Fe_3_O_4_ NPs and microfiber as shown in Figure 1c–f. The macroscopic images in Figure 1c–f illustrate that more composites were deposited near the magnet as the concentration of Fe_3_O_4_ NPs increased. The EDX mapping detected the C, O and Fe elements in Figure 1c–f indicated that the Fe element was attached to the fiber surface. As the concentration of Fe_3_O_4_ NPs increased, the distribution of Fe elements on the fiber surface increased. These results confirmed that the wood fiber could be used as matrix materials for Fe_3_O_4_ NPs.

**Figure 2:**
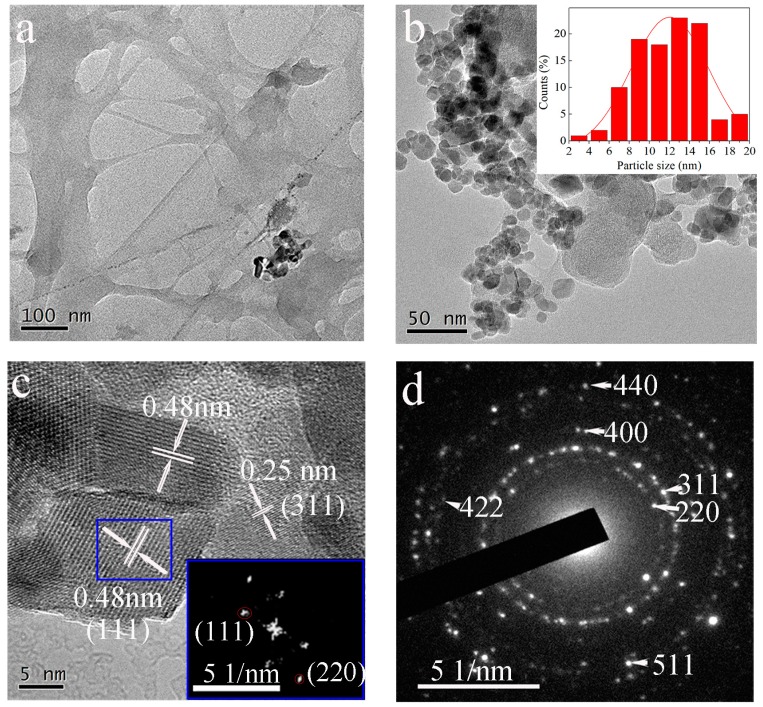
Transmission electron microscopy (TEM) images of treated fiberboard (**a**) and NFF composite board (**b**). The inset in (**b**) shows the particle distribution. The high resolution TEM (HRTEM) image (**c**) and selected area electron diffraction (**d**) of the NFF composite board, and the inset displays the electron diffraction of the blue area in (**c**).

Figure 2a,b shows the TEM images of the fiber and NFF composites. As can be seen in Figure 2a, the treated fiber was branched, and multi-scale filaments appeared near the fiber. The nanofibers were peeled from the fiber surface and connected together by physical absorbing [20], hydrogen bonding and adhesive combining. As can be seen in Figure 2b, the fiber surfaces were covered by Fe_3_O_4_ NPs, which could be prevented from agglomerating by mechanical stirring. Furthermore, Fe_3_O_4_ NPs were still attached to the fiber surface, even after ultrasound treatment, indicating that a superior adhesion between Fe_3_O_4_ NPs and fiber was obtained. As was shown in Figure 2b, parts of the NPs were cubic Fe_3_O_4_, and the size of crystal particles was less than 20 nm. The inset in Figure 2b shows the histogram of the particle distribution and the lognormal fitting curve of Fe_3_O_4_ NPs. Furthermore, the crystalline phase of the Fe_3_O_4_ NPs was obtained by high resolution TEM (HRTEM) as shown in Figure 2c. The distance of 0.48 nm was corresponded to the lattice distance of (111) planes; the lattice fringe spacing of 0.25 nm matched with that of (311) planes [21]. The inset shows the electron diffraction of the blue area in Figure 2c, and the diffraction spots of atoms are seen clearly. The selected area electron diffraction (SAED) pattern taken from NFF composites shows multiple diffraction rings in Figure 2d, corresponding to crystalline reflections of (220), (311), (400), (422), (511) and (400), respectively [21].

**Figure 3:**
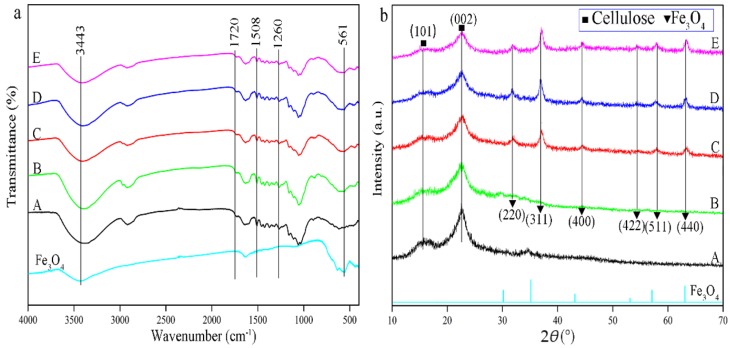
Fourier transform infrared spectroscopy (FTIR) spectra (**a**) and X-ray diffraction (XRD) patterns (**b**) of fiber pulp, nano-Fe_3_O_4_ and NFF composite pulp. (A. treated fiber; B. NFF1; C. NFF2; D. NFF3; E. NFF4).

The FTIR spectra of treated fibers, nano-Fe_3_O_4_ and NFF composites are displayed in Figure 3a. The peaks at 3443 cm^−1^ in the FTIR spectra were ascribed to O–H stretching, and corresponded to the surface-absorbed water and hydroxyl groups [22]. This result was ascribed to the hydroxyls of the fiber surface increasing after the wood fiber was treated. The absorption peaks at 2916 and 1372 cm^−1^ were ascribed to –CH_3_ stretching vibration and CH_2_ bending vibration, respectively. The absorption band at 1720 cm^−1^ corresponded to C=O stretching of hemicellulose [23]. The decrease in peak intensity at 1720 cm^−1^ was attributed to the hydrolysis of hemicellulose. The absorption band at 1630 cm^−1^ was assigned to absorbed water or C=C stretching vibration. The absorption peak at 1508 cm^−1^ was corresponded to the skeletal vibration of the aromatic ring, which derived from lignin [19]. The bands at 1260 and 1060 cm^−1^ were assigned to C–O–C (aryl-alkyl ether linkage) stretching vibration and C–O (alkoxy) stretching vibration, respectively [24]. The absorption peak at 561 cm^−1^ was attributed to Fe–O stretching vibration [25,26], which confirmed that the Fe_3_O_4_ NPs were mixed in the fiber. The characteristic peaks of fiber were not weakened or disappeared, whereas the characteristic peaks of metal-oxide became strengthened. The results indicated that Fe_3_O_4_ had no effect on the relative intensity of characteristic peaks of wood fibers.

Figure 3b shows the XRD patterns of cellulose, nano-Fe_3_O_4_ and NFF composites. The two diffraction peaks at 2*θ* = 22.8° and 16.2°, standing for (002) plane and (101) plane, respectively, which were the characteristic peaks of cellulose [27]. It can be observed that the peaks appeared at 2*θ* = 18.2°, 30.6°, 35.7°, 43.4°, 57.4°, and 62.9°, which stand for the (111), (220), (311), (400), (511) and (440) planes of the Fe_3_O_4_ (PDF No. 19-0629), respectively [28,29]. The characteristic peaks of nano-Fe3O4 and fiber were observed in the XRD patterns of nano-Fe_3_O_4_/fiber. It could be confirmed that the Fe_3_O_4_ NPs were mixed in the fiber. Combined with the results of SEM, TEM and FTIR, it could be concluded that the Fe_3_O_4_ NPs were loaded on the fiber surface. In particular, as the content of nano-Fe_3_O_4_ increased, the relative intensities of diffraction peaks significantly increased. The average crystalline size of nano-Fe_3_O_4_ particles on the fiber can be obtained by the Debye-Scherer equation [27]:

(1) d=Kλ/(βcosθ)

where *λ* was the wavelength of the radiation, K was 0.89, 2*θ* was the Bragg’s angle, and *β* was the full width at half maximum of (311) plane. The average crystalline size of the nano-Fe_3_O_4_ particles was approximately 18.2 nm. The results were greater than the values from the TEM calculation.

**Figure 4:**
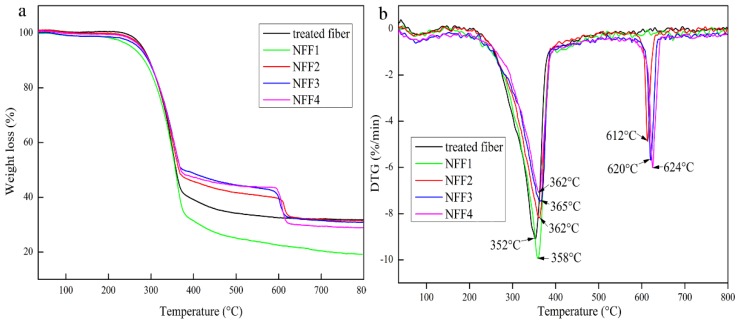
Thermogravimetric (TG) (**a**) and differential thermogravimetric (DTG) (**b**) curves of the fiber and NFF composites under an air atmosphere.

The thermogravimetric (TG) and differential thermogravimetric (DTG) curves of the fiber and NFF composites are displayed in Figure 4. In Figure 4a, a slight weight loss of 1–2% appeared in the range of 30–150 °C in all TG curves. This result was ascribed to the evaporation of the physically absorbed water in the wood fiber [30,31]. The pure fiber sample showed a step of weight loss in the range of 250–450 °C, and rapid weight loss was found at a temperature of 352 °C. This result was ascribed to the thermal decomposition and oxidation of the hemicellulose and cellulose. However, for NFF composites, the TG curves had an additional stage in the range of 550–650 °C. The weight losses of NFF2, NFF3 and NFF4 in the range of 550–650 °C were 7%, 10% and 13%, respectively, as the concentration of NPs increased. In Figure 4b, with the NPs content increasing, the first peak of rapid weight loss of composites was shifted to higher temperatures. As seen in Figure 4b, the first peak temperature of the pure fiber was 352 °C; the first peak temperatures of NFF1, NFF2, NFF3 and NFF4 were 358, 362, 365 and 362 °C, respectively. The second rapid weight loss of nanocomposites appeared approximately 620 °C, indicating the oxidation and decomposition of Fe_3_O_4_ in the air atmosphere. As was reported in previous literature, Fe^3+^ was reduced to Fe^2+^ in the char matrix, Fe^2+^ was further reduced to Fe^0^ at 580–650 °C [32]; the final product may be Fe.

**Figure 5:**
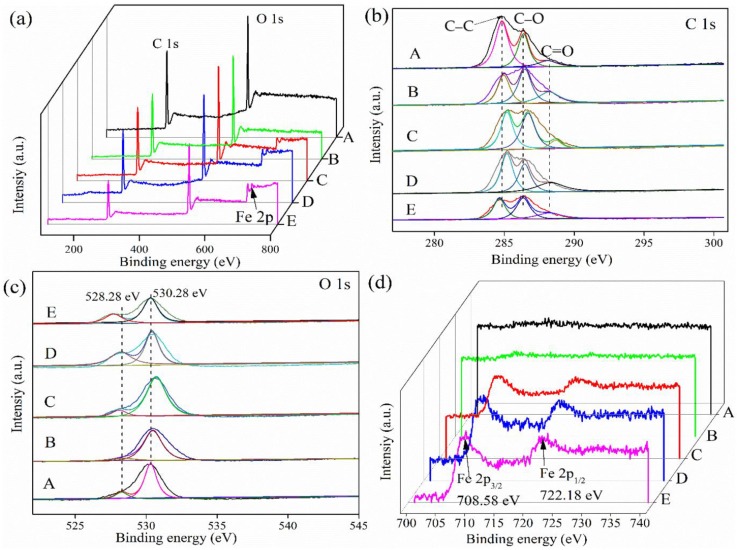
(**a**) X-ray photoelectron spectroscopy (XPS) survey spectra of treated fiber and NFF composite; High-resolution C 1s (**b**), O 1s (**c**) and Fe 2p (**d**) XPS spectra of fiber and NFF composite. (A. treated fiber; B. NFF1; C. NFF2; D. NFF3; E. NFF4).

The surface atomic composition and chemical bond of fibers and NFF composites are shown in Figure 5. Figure 5a exhibits the survey XPS spectra of fibers and NFF composites. Meanwhile, Table 1 listed the atomic percentage and C/O ratio. The Fe element was observed in the XPS spectra of the NFF composite, and the atomic percentages were 0.92%, 2.54%, 4.83% and 5.78%, respectively. The C/O ratio of fibers was 2.40, while that of NFF composites gradually decreased to 1.50. In Figure 5b, the C 1s XPS spectra of the fibers was divided into three peaks with binding energies of 284.88, 286.28 and 288.28 eV belonging to C–C, C–O and C=O bonds, respectively [33]. The relative intensity of C–C and C–O of NFF composites decreased as the content of NPs increased compared with that of the fiber. As shown in Figure 5c, the O 1s binding energies of the fibers were approximately 528.28 and 530.28 eV, which can be ascribed to water absorption and OH groups [34]. For NFF composites, the peak at 528.28 eV shifted to the low binding energy bond at 527.68 eV, which assigned to the metal-oxygen bond. The peak showed that the iron element was attached on the fiber surface by Fe–O bonding [25,29]. As shown in Figure 5d, the Fe 2p spectra of the NFF composite was divided into two sharp peaks at 708.58 and 722.18 eV, which linked to Fe 2p_3/2_ and Fe 2p_1/2_ spin-orbit peaks, respectively [35,36]. The satellite peak of Fe 2p became more obvious as the content of Fe_3_O_4_ NPs increased. The satellite peak of Fe 2p_3/2_ was located approximately 8 eV higher than the main Fe 2p_3/2_ peak. The satellite peak that appeared can be supported the deposition of Fe_3_O_4_ NPs in NFF composites.

**Figure 6:**
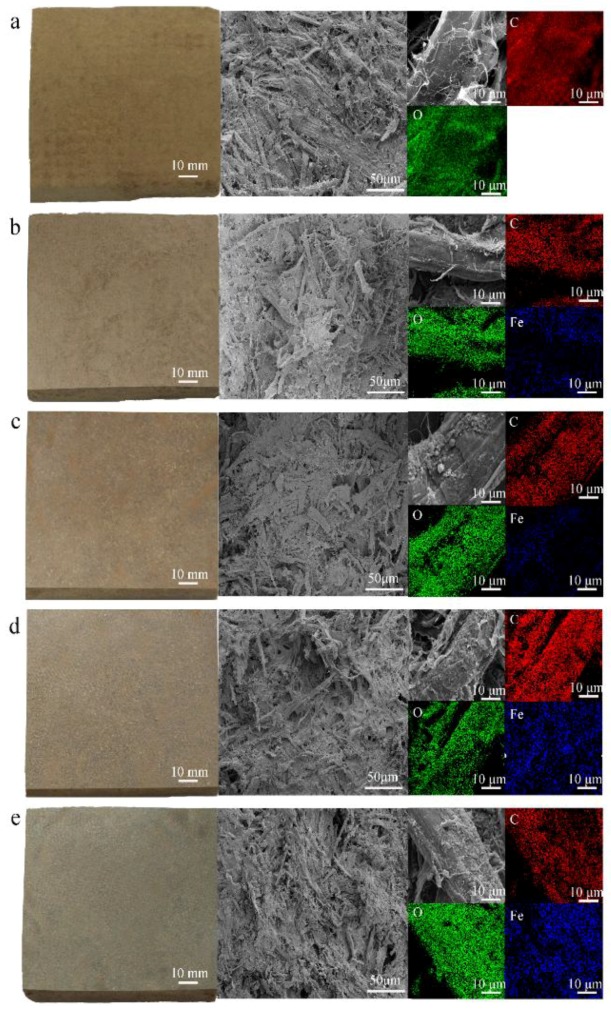
The digital images, SEM and EDX mapping images of pure binderless fiberboard and NFF binderless boards. (**a**) fiberboard; (**b**) NFF1 board; (**c**) NFF2 board; (**d**) NFF3 board; (**e**) NFF4 board.

Figure 6 shows the digital images, SEM and EDX elemental mapping images of all binderless board samples. Figure 6a displays the macroscopic morphology of the pure binderless fiberboard; the surface and sections were still rough after surface modification. As shown in SEM images, the fiber surface appeared such as a microfiber. At high magnification, multi-scale filaments distributed on the surface of fiber and part filaments were linked to form a sheet structure. EDX detected the composition of the binderless fiberboard; the main elements were C and O. Compared with the macro-morphology of pure fiberboard, after surface modification, NFF binderless composite boards had a smooth surface and sections in Figure 6b–e; the surface color of NFF composite boards deepened. The fibers of the NFF board were cross-linked by microfibers in the SEM image of Figure 6b–e. Under 5000× magnification, Fe_3_O_4_ NPs and microfibers were observed on the fiber surface. The EDX elemental mapping showed that C, O, Fe elements were distributed on the fiber structure, which was further confirmed by Fe_3_O_4_ deposited on the NFF board. As the concentration of Fe_3_O_4_ NPs increased, the distribution of Fe elements widen.

**Figure 7:**
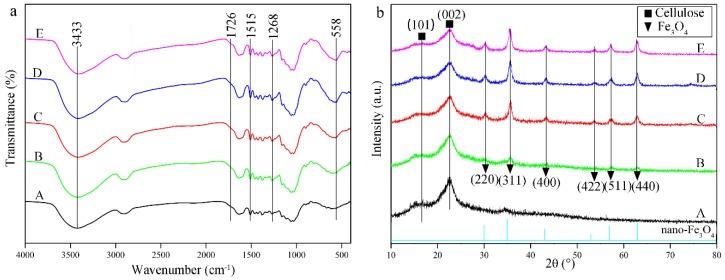
FTIR spectra (**a**) and XRD patterns (**b**) of binderless fiberboard and NFF binderless composite boards. (A. fiberboard; B. NFF1 board; C. NFF2 board; D. NFF3 board; E. NFF4 board.)

The FTIR spectra of binderless fiberboard and NFF binderless composite boards are displayed in Figure 7a. The OH stretching of NFF binderless composite boards was detected at 3343 cm^−1^, less than the absorbing peak position of the NFF composite (Figure 3a). The absorption peak of OH stretching increased to the low wavenumber, indicating that hydroxyl could form hydrogen bonds during hot-pressing. Contrasted with the composite pulp (Figure 3a), the characteristic peak of hemicellulose at 1726 cm^−1^ weakened [22], indicating that the chemical structure of hemicellulose changed. The peak at 1515 cm^−1^ corresponded to the skeletal vibration of the aromatic ring, which derived from lignin [19]. The bands at 1268 and 1060 cm^−1^ were ascribed to the C–O–C (aryl-alkyl ether linkage) stretching vibration and C–O (alkoxy) stretching vibration, respectively [24,37]. As the concentration of Fe_3_O_4_ increased, the transmittance of these characteristic peaks did not change. The Fe–O stretching vibration at 558 cm^−1^ indicated that Fe_3_O_4_ NPs were deposited into NFF binderless composite boards [25,26]. As the concentration of Fe_3_O_4_ NPs increased, the characteristic peaks became sharper.

Figure 7b shows the XRD patterns of binderless fiberboard and NFF binderless composite board. For the NFF binderless composite board, the peaks at 2*θ* = 16.7° and 22.6° corresponded to the (101) and (002) planes of cellulose [25]. The diffraction peaks at 18.4°, 30.3°, 35.4°, 43.5°, 53.8°, 57.5°, and 62.8° corresponded to the (111), (220), (311), (400), (422), (511) and (440) planes of the Fe_3_O_4_ (PDF No. 19-0629 [2]), respectively [28]. It could be confirmed that the nano-Fe_3_O_4_ were deposited on the fiber surface. As the concentration of nano-Fe_3_O_4_ increased, the diffraction peak intensities of Fe_3_O_4_ significantly increased, but the relative intensities of the cellulose diffraction peak (002) decreased.

**Figure 8:**
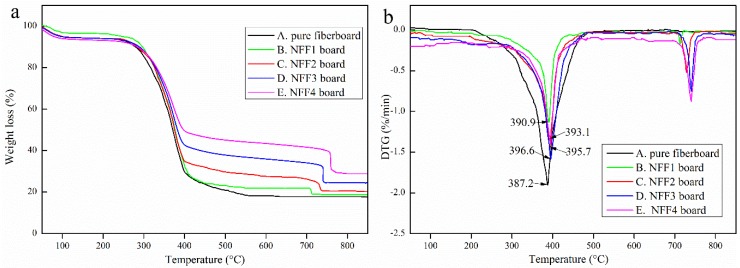
TG (**a**) and DTG (**b**) curves of binderless fiberboard and NFF binderless composite board. (A) pure fiberboard; (B) NFF1 board; (C) NFF2 board; (D) NFF3 board; (E) NFF4 board.

Figure 8 shows the TG and DTG curves of binderless fiberboard and NFF binderless composite board. The TG curves of these samples could be separated into three parts. The weight loss below 150 °C could be attributed to the evaporation of water [30,31]. Then, a significant weight loss appeared between 250 and 450 °C, which was attributed to the thermal decomposition of the wood fiber composition. However, for the NFF binderless composite board, a significant weight loss appeared in the third stage between 700 and 800 °C, which was ascribed to the thermal decomposition and oxidation of metal oxide. The binderless fiberboard displayed rapid weight loss at a temperature of 387.2 °C, which was higher than that of fiber pulp. The result could be interpreted as hydrogen bond and covalent bond appearing due to hot-pressing, which would improve the thermal stability of the binderless fiberboard. As the content of Fe_3_O_4_ increased, the thermal degradation temperature of the composite board increased. The temperature of the first degradation stage was 387.2, 390.9, 393.1, 395.7 and 396.6 °C, respectively. Combined with the data analysis of the fiber pulp, it could be concluded that Fe_3_O_4_ NPs had an effect on thermal stability of binderless composite board.

**Figure 9:**
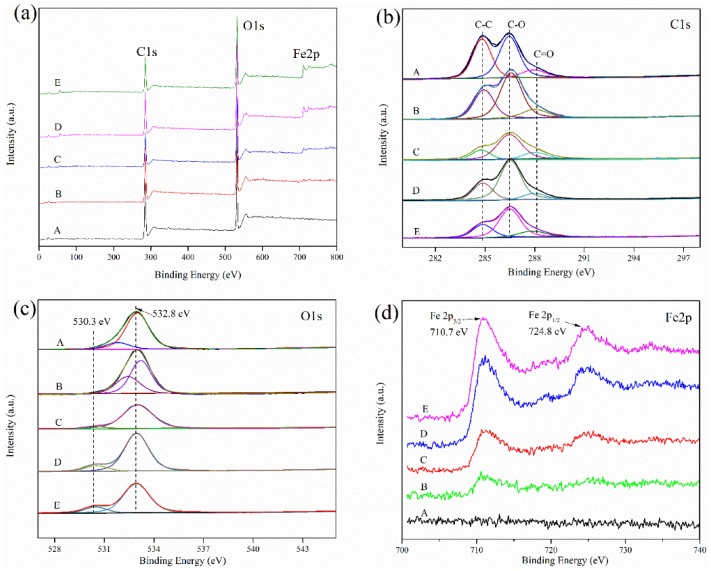
(**a**) XPS survey spectra; (**b**) C 1s XPS spectrum; (**c**) O 1s XPS spectrum and (**d**) Fe 2p XPS spectrum of binderless fiberboard and NFF binderless composite board. (A. fiberboard; B. NFF1 board; C. NFF2 board; D. NFF3 board; E. NFF4 board).

The atomic compositions of binderless fiberboard and NFF binderless composite board are displayed in Figure 9. Figure 9a showed the XPS survey spectra of the binderless fiberboard and NFF binderless composite board, and the data are shown in Table 2, which were obtained using XPS analysis. The Fe element was detected in the NFF binderless composite board; the atomic percentages were 0.97%, 1.95%, 2.45%, and 3.68%. The C/O ratio of pure binderless fiberboard was 2.17, while the C/O ratio of NFF binderless composite boards were 1.87, 1.61, 1.46, and 1.38. It was confirmed that the Fe element had an effect on the C/O ratio of the binderless composite board. Figure 9b shows the C 1s XPS spectra. It was divided into three peaks at binding energies of 284.8, 286.5, and 288.2 eV, which were ascribed to C–C, C–O and C=O bonds, respectively [38]. As the content of nano-Fe_3_O_4_ increased, the relative intensities of C–C and C–O peaks decreased. As shown in Figure 9c, the O 1s fitting peaks of pure binderless fiberboard at 531.7 and 532.8 eV were ascribed to absorbed water and oxygen in OH groups, respectively [39]. Compared with the NFF composite, the O 1s band of the NFF binderless composite board was shifted to high binding energy. This result indicated that the binding energy of O 1s increased as the electron density of oxygen atoms decreased. For the NFF composite board, the O 1s had an extra peak at 530.3 eV, which corresponded to the metal-oxygen bond [25]. The high-resolution Fe 2p XPS spectra of the NFF binderless composite board is displayed in Figure 9d. The XPS spectrums exhibited two sharp peaks at 710.7 and 724.8 eV, which were ascribed to Fe 2p_3/2_ and Fe 2p_1/2_ spin-orbit peaks, respectively [40,41]. Compared with the XPS spectrum of the NFF composite, the atomic percentage of Fe elements in the composite board was less than that of the mixed composite, and the C/O ratio was less than that of the mixed composite.

**Figure 10:**
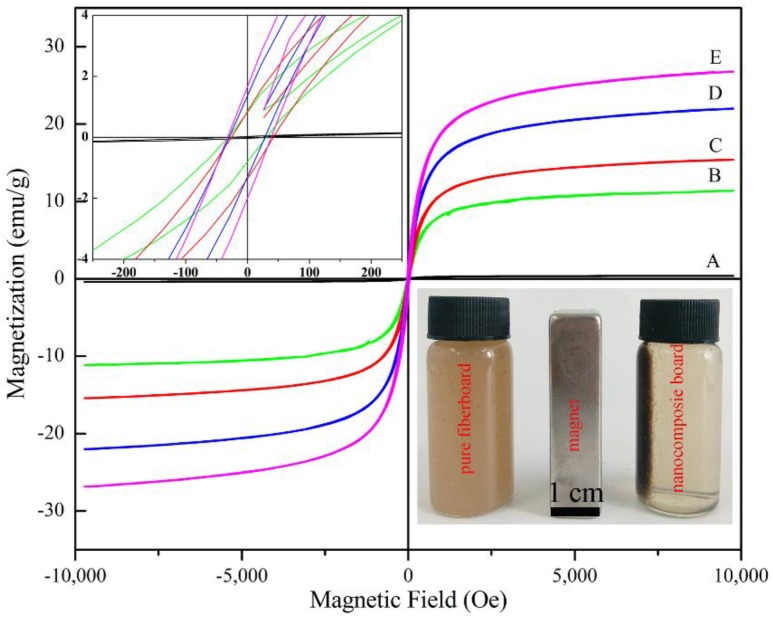
Magnetic hysteresis loops of binderless composite board with different mass fractions. The top left inset shows an enlarged view of the magnetic hysteresis loops. The bottom right inset shows the magnetic response under an external magnetic field. (A. pure fiberboard; B. NFF1 board; C. NFF2 board; D. NFF3 board; E. NFF4 board).

Figure 10 displays the magnetic properties of NFF binderless composite board by measuring their magnetization curves. The NFF binderless composite board with 1%, 3%, 6%, and 9% iron content exhibited saturation magnetizations of 11.03, 15.39, 21.89, and 26.78 emu/g, respectively. The data indicated that the maximum saturation magnetization was 26.78 emu/g, which was lower than that of pure Fe_3_O_4_ nanocrystals [42] when the content of Fe_3_O_4_ NPs was 9%. As shown in the top left inset, the enlarged hysteresis displayed low coercivity of 80 Oe. The low coercivity determined that the materials were magnetically soft and could convert polarity [4,43]. When the magnet was placed beside two bottles filled with NFF composite and pure binderless fiberboard powder, the NFF composite quickly moved along the magnetic field and the pure fiberboard did not change. The experimental results indicated that the magnetism of binderless composite boards was derived from Fe_3_O_4_ NPs. The pure Fe_3_O_4_ was a superparamagnetic material, and it had high saturation magnetization (*M*_s_), remnant magnetization (*M*_r_) and low coercivity (*H*_c_) [44]. Therefore, the saturation magnetization and remnant magnetization of composite board increased as the content of Fe_3_O_4_ NPs increased.

The electromagnetic absorption properties of NFF binderless composite boards were tested by mixing 20 wt % samples with paraffin. The reflection loss (RL) curves were obtained by the following equations [45]:

(2) $$Z_{\mathrm{in}}=Z_{0}\sqrt{\frac{\mu_{\gamma }}{\varepsilon_{\gamma }}}\tanh\left[j\left(\frac{2\pi fd}{c}\right)\sqrt{\mu_{\gamma }\varepsilon_{\gamma }}\right]$$

(3) $$\mathrm{RL}=20\log\frac{Z_{\mathrm{in}}-Z_{0}}{Z_{\mathrm{in}}+Z_{0}}$$

where *μ_γ_* and *ε_γ_* was the relative complex permeability and the complex permittivity, respectively, Z_0_ was the impedance of free space, Z_in_ was the input impedance, *f* was the microwave frequency, *d* was the absorber thickness, and c was the velocity of the electromagnetic waves.

**Figure 11:**
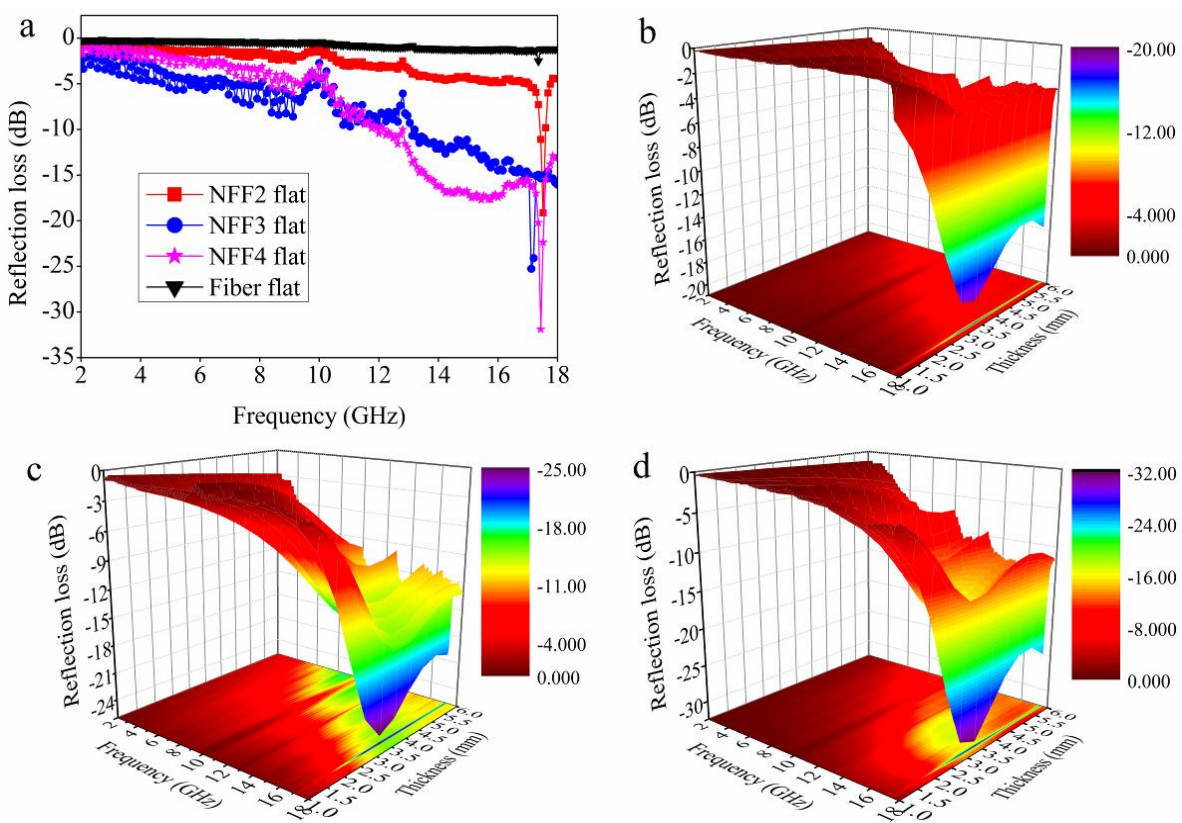
(**a**) Reflection loss curves of NFF composites at a frequency range of 2–18 GHz with a thickness of 3.5 mm; The three-dimensional graph of reflection loss of NFF2 board (**b**); NFF3 board (**c**) and NFF4 board (**d**).

Figure 11a reveals the RL curves of pure binderless fiberboard and NFF binderless composite board. The reflection loss of pure fiberboard had a minimum value of −2.51 dB. The RL values of the NFF2, NFF3 and NFF4 boards were −19.13, −25.26 and −31.90 dB at 17.52, 17.12 and 17.44 GHz, respectively. The RL value of −10 dB corresponded to 90% EM absorption [21]. The minimum RL values of NFF3 and NFF4 were less than −10 dB in the ranges of 13.2–18.0 and 12.4–18.0 GHz, respectively. Figure 11b–d shows the three-dimensional RL images of the NFF2, NFF3 and NFF4 boards, revealing the influence of thickness and frequency on the absorption properties. The RL value of the NFF4 board in a range of 17.36–17.52 GHz was less than −20 dB, and the minimum value was up to −31.90 dB at 17.44 GHz, which was higher than EM absorbing results of other common absorbing materials [5,46]. The enhanced microwave absorption properties were ascribed to the combination of complex permittivity, permeability and dielectric loss [47]. Therefore, the results demonstrated that the deposition of Fe_3_O_4_ NPs improved the microwave absorbing properties of the NFF binderless composite board.

**Figure 12:**
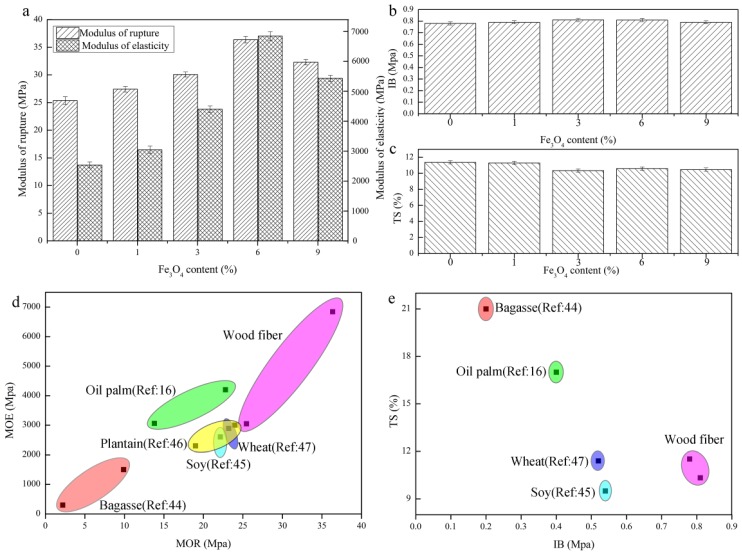
(**a**) The histogram of the modulus of rupture and modulus of elasticity of pure binderless fiberboard and NFF board; the histogram of internal bond (IB) strength (**b**) and thickness swelling (TS) rate (**c**) of pure fiberboard and NFF composite boards with different contents of Fe_3_O_4_ NPs; (**d**,**e**) Comparison of the mechanical properties of NFF composite board and other biomass composites.

Figure 12a shows the modulus of rupture (MOR) and modulus of elasticity (MOE) of the pure binderless fiberboard and magnetized binderless composite board. As shown in the histogram, the MOR and MOE of pure binderless fiberboard were 25.35 and 2537.59 MPa, respectively. As the Fe_3_O_4_ content increased, the MOR and MOE of binderless composite board reached the maximum values of 36.36 and 6842.16 MPa, respectively. Compared with the pure binderless fiberboard, the MOR and MOE of the binderless composite board increased by 43.4% and 169.6%, respectively. This result was attributed to the synergistic enhancement of hydrogen bond and nanoparticles. The rigid Fe_3_O_4_ NPs were dispersed on the fiber matrix by hydrogen bonding and physical cross-linking; the external load can be effectively transferred to the rigid particles by the interface connection layer. However, the MOR and MOE of binderless composites decreased as the content of Fe_3_O_4_ NPs increased to 9%. This result was ascribed to the undesirable stress concentration points generated as the content of Fe_3_O_4_ increased. Figure 12b shows the internal bond (IB) strength of pure binderless fiberboard and NFF binderless composite board. The value of internal bond strength of pure binderless fiberboard was 0.78 MPa. As the content of Fe_3_O_4_ NPs increased, the IB strength was 0.81 MPa. However, as the content of Fe_3_O_4_ NPs reached 9%, the IB value was 0.79 MPa. As the Fe_3_O_4_ NPs of the fiber surface reached a certain quantity, the agglomeration of NPs disturbed the IB strength. Figure 12c displays the thickness swelling (TS) values of pure binderless fiberboard and NFF binderless composite board. All TS values less than 20%; the values varied from 10.34% to 13.36%. With the addition of Fe_3_O_4_ NPs, the TS values tended to decrease. This result may be related to densification of the surface by Fe_3_O_4_ NPs covering the fiber surface, and the Fe_3_O_4_ NPs disturbed the bond between hydroxyls of cellulose and water. Figure 12d,e shows the mechanical properties of NFF binderless composite board and that of other peer biomass materials, which were greater than other materials [17,48,49,50,51].

## 4. Conclusions

In summary, NFF binderless composite boards were prepared by hot-pressing after being pretreated by a green mechanical method. The Fe_3_O_4_ NPs were distributed on the surface of the fiber matrix. The as-prepared Fe_3_O_4_/Fiber binderless composite board not only had microwave absorption properties but also possessed good mechanical properties. The maximum saturation magnetization of the composites was 26.78 emu/g with low coercivity of 70 Oe. The minimum reflection loss was −31.90 dB at 17.44 GHz for the NFF4 board with a thickness of 3.5 mm. The NFF binderless composite board had a strong MOR of 36.36 MPa, an MOE of 6842.16 MPa and an IB of 0.81 MPa; the values were ascribed to the rigid Fe_3_O_4_ NPs enhancing the mechanical strength. As Fe_3_O_4_ NPs were added, the TS reached a minimum value of 10.34%. This study showed that the good mechanical properties and microwave absorbing performance of the 

NFF binderless composite boards were attributed to the synergetic enhancement of nano-Fe_3_O_4_ and fiber. The boards can be applied to indoor furniture to reduce the harm of EM interference.

## Figures and Tables

**Table 1:** Element content and C/O atomic ratio of fiber and nano-Fe_3_O_4_/Fiber (NFF) composite.

Sample	C (at %)	O (at %)	Fe (at %)	C/O Ratio
Fiber	68.63	31.37	-	2.40
NFF1	67.33	31.75	0.92	2.12
NFF2	63.64	33.82	2.54	1.88
NFF3	61.59	33.58	4.83	1.83
NFF4	56.55	37.67	5.78	1.50

**Table 2:** Element content and C/O ratio of binderless fiberboard and NFF binderless composite board.

Sample	C (at %)	O (at %)	Fe (at %)	C/O ratio
Fiberboard	68.48	31.52	-	2.17
NFF1 board	64.53	34.5	0.97	1.87
NFF2 board	60.55	37.5	1.95	1.61
NFF3 board	57.87	39.68	2.45	1.46
NFF4 board	55.93	40.39	3.68	1.38
